# Optical Read-out of Neural Activity in Mammalian Peripheral Axons: Calcium Signaling at Nodes of Ranvier

**DOI:** 10.1038/s41598-017-03541-y

**Published:** 2017-07-18

**Authors:** Arjun K. Fontaine, Emily A. Gibson, John H. Caldwell, Richard F. Weir

**Affiliations:** 1 Department of Bioengineering, University of Colorado – Anschutz Medical Campus, Colorado, USA; 2 Department of Cell and Developmental Biology, University of Colorado – Anschutz Medical Campus, Colorado, USA

## Abstract

Current neural interface technologies have serious limitations for advanced prosthetic and therapeutic applications due primarily to their lack of specificity in neural communication. An optogenetic approach has the potential to provide single cell/axon resolution in a minimally invasive manner by optical interrogation of light-sensitive reporters and actuators. Given the aim of reading neural activity in the peripheral nervous system, this work has investigated an activity-dependent signaling mechanism in the peripheral nerve. We demonstrate action potential evoked calcium signals in mammalian tibial nerve axons using an *in vitro* mouse model with a dextran-conjugated fluorescent calcium indicator. Spatial and temporal dynamics of the signal are presented, including characterization of frequency-modulated amplitude. Pharmacological experiments implicate T-type Ca_V_ channels and sodium-calcium exchanger (NCX) as predominant mechanisms of calcium influx. This work shows the potential of using calcium-associated optical signals for neural activity read-out in peripheral nerve axons.

## Introduction

The present work addresses the need for improved neural interfacing strategies by investigating a novel mechanism for neural read-out. The activity-dependent calcium signaling characterized here facilitates an optogenetic approach to meet this challenge by enabling minimally invasive optical communication with specific (individual) nerve fibers.

Sophisticated prostheses such as artificial hands^1, 2^ have the mechanical capability to largely substitute functionality in many biological systems. The largest barrier preventing true limb replacement is the lack of an adequate neural interface that enables full and intuitive control of prosthetic devices. Without this communication to the nervous system, artificial limbs and their control remain limited, capable of only crude movements and lacking sensory feedback. State-of-the-art surface electromyographic (EMG) control lacks the ability to communicate with small or deeply embedded muscles for single digit command or fine-motor control, and offers no means of sensory feedback.

The peripheral nerve offers a logical location to interface because simplified neural outputs of complex CNS circuitry are contained in a spatially concentrated bundle. A peripheral nerve interface for neuroprosthetic and therapeutic applications would require minimally invasive axon communication with high specificity in order to achieve precise neural read-out (i.e. individual digit motor command) and well-defined afferent read-in (i.e. localized tactile feedback). Electrode-based nerve interfaces^3–6^ do not meet these criteria, due predominantly to insufficient specificity and difficulty deciphering minute electrical signals within a nerve from peripheral electrodes.

The recently emergent field of optogenetics offers a unique set of tools that may offer an improved solution to the peripheral nerve interface. By expressing light-sensitive opsins in specific cell types, it is feasible to optically query neural signals for motor read-out as well as stimulate activity for sensory read-in for targeted axons within a nerve bundle. In contrast to electrode-based techniques of neural recording/stimulation, which cannot decipher signals from single neural pathways (rather they provide crude aggregate information from many units), optical interrogation can *specifically* query many single neurons or neuron processes because the spatial resolution of modern optical systems is well within the size of these features. Stimulation of nerve fibers can be achieved optically with the widely used ChannelRhodopsin2 (ChR2) or ChR2 variant, which has been used to stimulate peripheral nerve axons under activation via an optical cuff  ^7, 8^.

For read-out, neural activity can be monitored by optically detecting fluorescence transients coupled to action potentials. Genetically encoded voltage indicators (GEVIs) have developed greatly in recent years^9–11^ but still face major limitations in their ability to detect measurable *in vivo* signals. Sensitivity, dynamic range, and signal-to-noise ratio of GEVIs are currently surpassed by calcium-sensitive fluorescent proteins such as GCaMP. While GCaMP has demonstrated its ability to transduce action potential information into a fluorescence signal, it is important to note that neuronal GCaMP signals have exclusively been reported from the soma and dendrites. It is well established that activity-dependent calcium transients occur here, as well as in axon terminal boutons, but there is less literature showing intracellular calcium perturbation along the nerve (axon) itself. Chiu *et al*. have shown action potential induced calcium influx in rodent CNS (optic nerve) axons^12^, occurring in a uniform fashion over the axon. The same laboratory revealed calcium signaling occurring in the frog sciatic nerve, in a coupling with mitochondrial motility^13^, and Grundemann and Clark reported action potential-dependent calcium influx in CNS Purkinje cell axons, occurring locally at nodes of Ranvier^14^. Stimulation-evoked calcium in small diameter unmyelinated axons of the autonomic nervous system have also been reported^15, 16^. However, until very recent work^17^ done in parallel with the research of our laboratory, there had been no published data showing activity-dependent calcium transients in myelinated axons of the mammalian peripheral nervous system. Zhang & David^17^ characterized calcium responses to prolonged stimuli, while the present study considers a wide range of shorter duration signals which extend from single action potentials to steady-state trains. The experimental design of our study also allowed many axons of varying size to be monitored within the same nerve preparation, whereas Zhang & David were limited to single large diameter axons. These differing aspects provide additional physiological relevance to the use of these signals for neural interfacing.

The overall goal of this investigation was to identify and characterize a physiological signal that is amenable to single axon read-out in an optogenetic neural interface. The presence of activity-dependent calcium transients in peripheral nerve axons was explored using an *in vitro* mouse model with a synthetic calcium indicator. Action potential elicited calcium transients are among the first demonstrated in mammalian peripheral nerve axons, and signals were detected in response to single action potentials. We sought to characterize the dynamics of such signaling with respect to stimulus parameters in order to evaluate its utility for neural interfacing purposes. Additionally, we aimed to determine the primary sources and mechanisms of the activity-dependent calcium signal.

## Results

### Activity-dependent calcium transients at nodes of Ranvier

**Figure 1:**
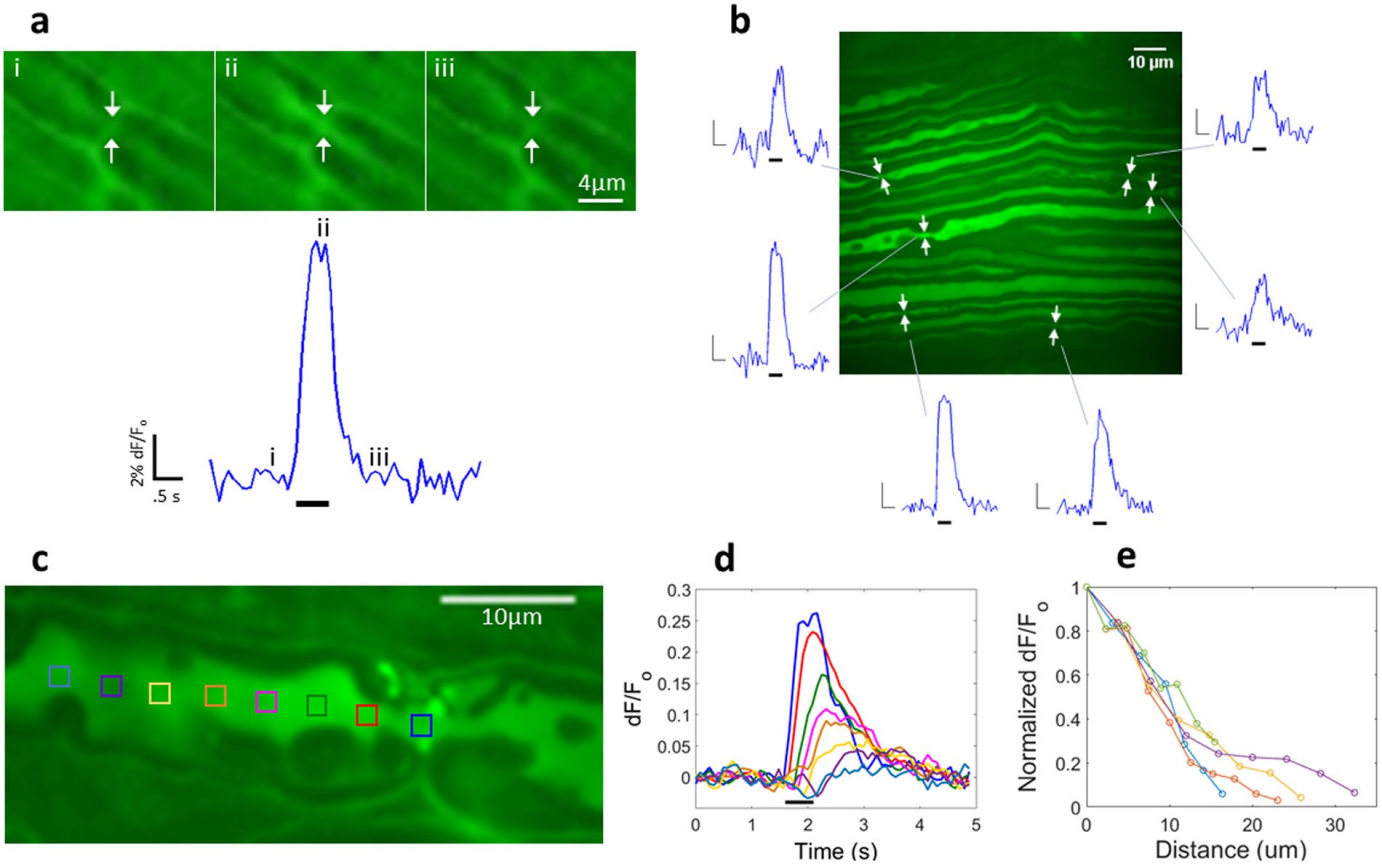
Single sweep action potential elicited calcium-responses at nodes of Ranvier (**a**) Response signal to 50 action potentials (100 Hz). Top panel images show an individual node of Ranvier before the action potential stimulus (*i*), during (*ii*), and after (*iii*) (arrows indicate the node location). Bottom panel is the quantitative optical signal produced by nodal image pixels (ROI is approximately 3 × 2 µm). Thick black bar indicates action potential stimulus. (**b**) Field of tibial nerve axons with at least six nodes of Ranvier yielding a calcium-coupled fluorescence change in response to a 1 s train of action potentials (100 Hz). Signal amplitudes among the six nodes range from 11–24%. *Inset scale bars:* 1 *s and* 5*% signal change*. (**c**) Longitudinal calcium signal propagation in an axon segment with node of Ranvier) (**d**) Activity dependent calcium fluorescence in response to a 50 action potential burst (100 Hz) recorded at incremental distances away from the node epicenter (signal trace colors correspond to ROIs in panel c). Plotted signals illustrate the latency in signal onset as well as reduced signal amplitude with distance from the node. (**e**) Peak normalized signal amplitude versus distance from node from five axon nodes. Yellow trace corresponds to the nodal data of panels c and d.

Action potential evoked calcium elevation was observed at axon nodes of Ranvier. The calcium-associated fluorescence is visually apparent (Fig. 1a and best visualized in Supplementary Video 1) and quantitative signal traces illustrate the sharp increase in fluorescence at the onset of stimulus, and abrupt decay upon action potential cessation. It was typical to observe multiple nodes of Ranvier responding to action potential stimulation in the same confocal field of view (Fig. 1b). The calcium signal originates at the node center and propagates distally and proximally into the internode. The maximum signal amplitude diminishes as a function of distance from the node epicenter while latency of signal onset increases (Fig. 1c–e). The signal amplitude follows a quasi-exponential fall-off with distance, as data from five axons was fit slightly better with an exponential function than with a linear fit (R^2^ = 0.949 and 0.934 respectively). Fluorescence data was used to calculate mean calcium wave propagation velocity (33.5 ± 6.1 µm/s) and spatial length constant (11.8 ± 1.0 µm) for a typical half-second long action potential burst applied in these experiments (50 APs, 100 Hz, n = 5).

### Signal modulation by number of action potentials

**Figure 2:**
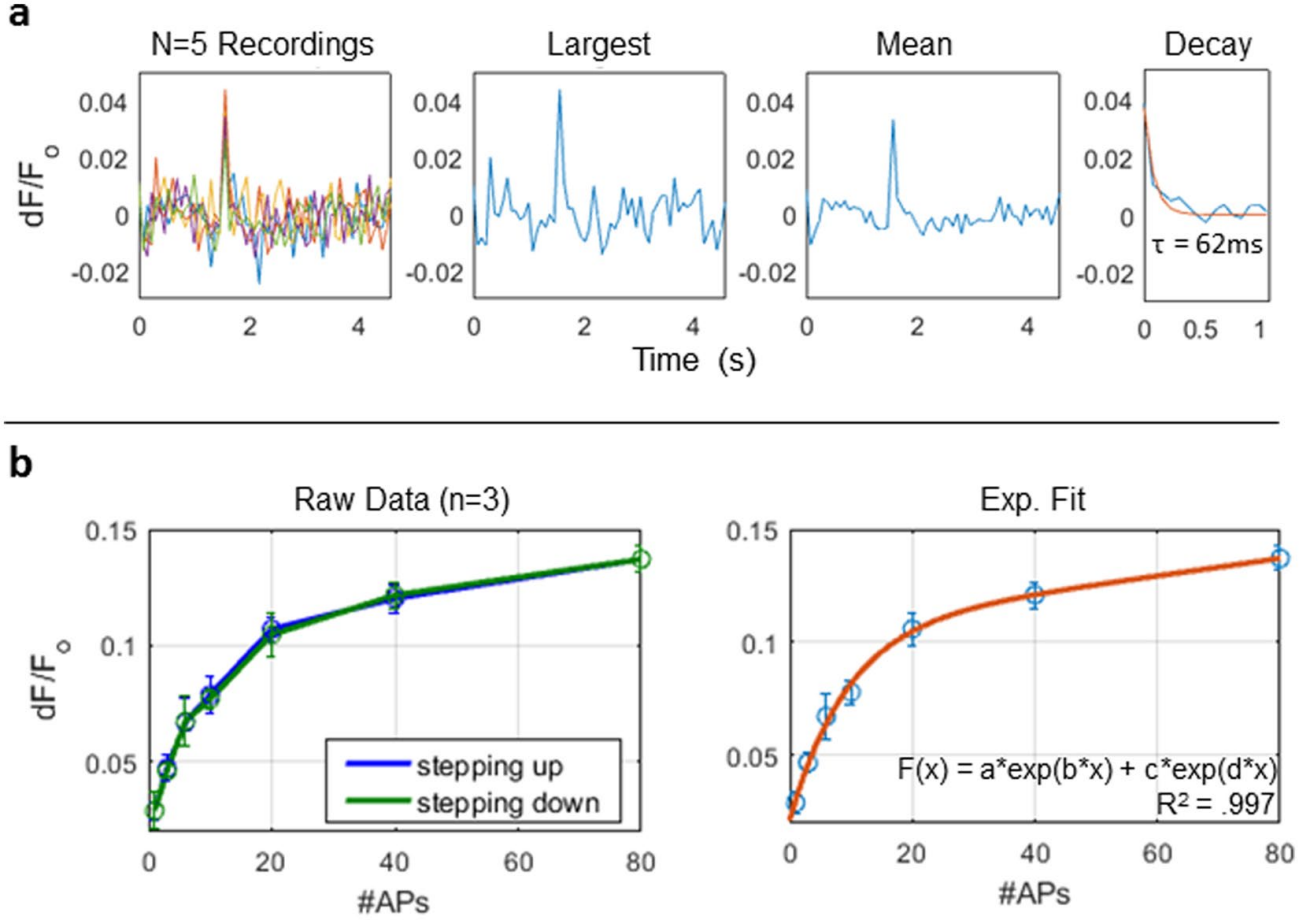
Calcium response to a single action potential and short bursts of action potentials. (**a**) Fluorescence response to a single action potential at a node of Ranvier, showing from left to right: five raw recordings, largest amplitude response trace, mean signal, and mean signal decay with exponential fit. (**b**) Mean fluorescence amplitude change with increasing number of action potentials in a stimulus train: a 100 Hz pulse train was applied for increasing durations to include more action potentials (1–80 APs). The signal amplitude begins to plateau as it reaches a relative steady state. Panel (left) shows mean amplitude measurements for three nodes acquired as the number of action potentials is ramped ‘up’ as well as ‘down’, showing no hysteresis. Panel (right): mean amplitude measurements are well fit by a double exponential function (R^2^ 0.997).

Changes in calcium fluorescence were detected in response to a single action potential (2.8% ± 0.5, n = 3 nodes) (Fig. 2a). As the number of action potentials in a stimulus is increased, the fluorescence amplitude increases accordingly. However, given constant action potential frequency, as the number of action potentials in a pulse train is increased, the signal amplitude will begin to plateau as it reaches a relative steady state for that frequency (Fig. 2b). The steady state saturation in this curve represents a balancing of calcium extrusion with influx, such that additional action potentials do not significantly change the amplitude. Additionally, amplitude data was collected as the number of action potentials was ramped up and down, to investigate whether ‘hysteresis’ takes place in this process. As the data show, no significant hysteresis-like effect was observed, as the amplitudes of the ramp up and down did not deviate.

### Signal modulation by action potential frequency

**Figure 3:**
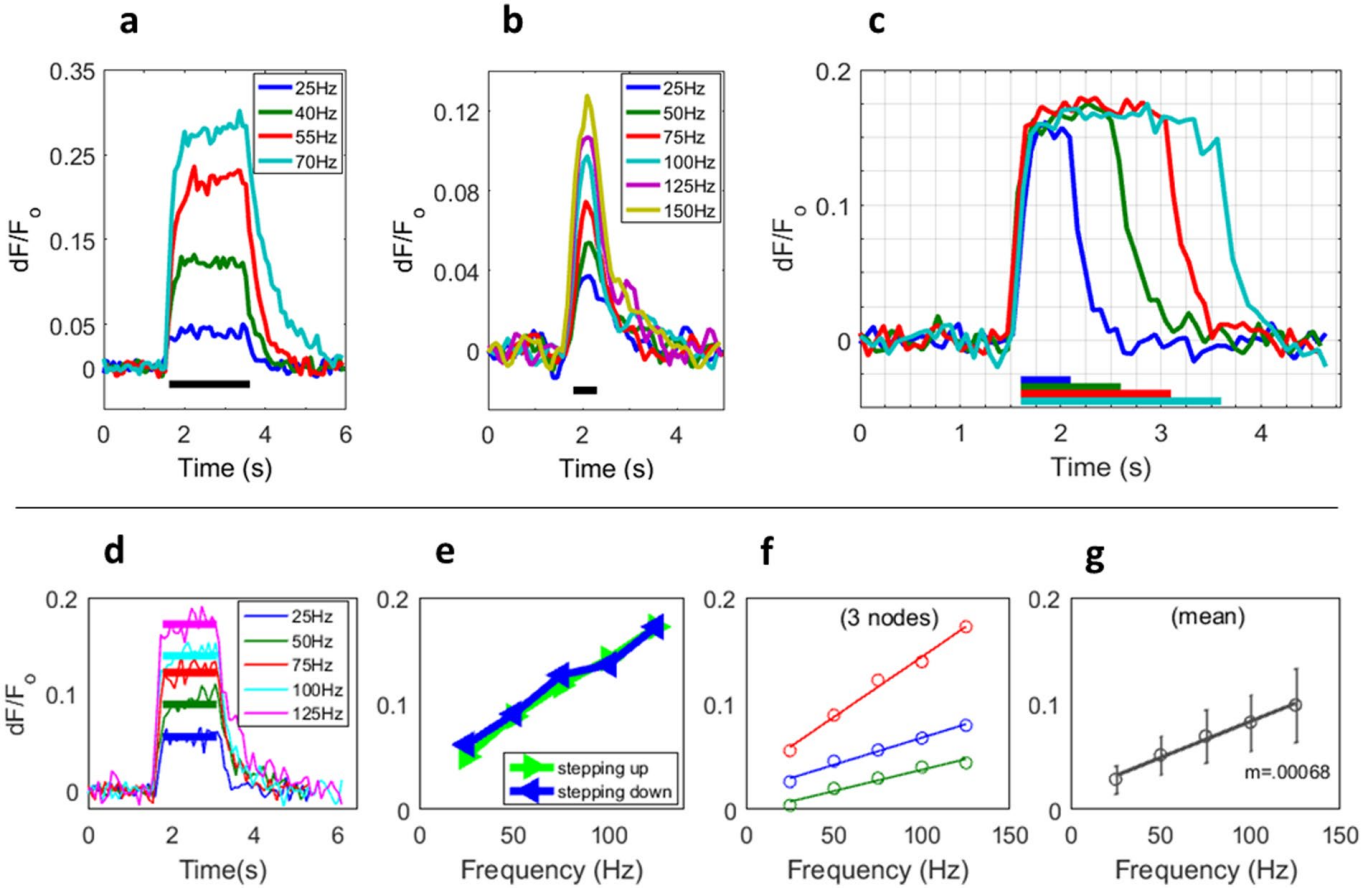
Activity-dependent calcium signal amplitude is modulated by action potential frequency and train duration. (**a**) Calcium signals in response to a 2 s stimulus, and (**b**) to a 0.5 s stimulus at a range of action potential frequencies (**c**) Calcium-fluorescence signals modulated by action potential train duration. Constant frequency (125 Hz) action potential trains are applied at 0.5, 1, 1.5 and 2 s (colored bars, bottom) and the calcium response duration closely follows the stimulus while preserving steady amplitude. The average decay constant for the four signals is 205 ± 15 ms (**d**) Frequency-modulated calcium fluorescence traces with bars indicating mean steady-state amplitude. (**e**) Stimuli/recordings were performed while stepping action potential frequency up and down showing no significant hysteresis effect. (**f**) Signal amplitude versus frequency of nodes from three independent nerve samples as recorded in panel d, and their mean (**g**) illustrates linear dependence with a slope of 0.07% fluorescence/Hz. Data shown are from single sweep recordings with the exception of panel f in which each trace is the mean of the up and down sweeps, and panel g which contains the sample mean.

In addition to this correlation between signal amplitude and number of action potentials, the dependence of the calcium signal amplitude on stimulus *frequency* was characterized. Nodal calcium responses to action potential trains of varying frequencies were tested for relatively long pulse trains of 2 s down to shorter bursts of 0.5 s. Frequencies of physiological range (≤150 Hz) graded the calcium fluorescence signal (Fig. 3) and the data showed a linear relationship between amplitude and frequency. For 2 s stimuli, signals were recorded for frequencies between 25–70 Hz as this would be a typical range of firing for a relatively sustained motor output. However, for shorter bursts, motor neurons are capable of firing significantly faster, up to approximately 150 Hz. As such, the responses to shorter stimuli were recorded with frequencies ranging from 25–150 Hz. The range of frequencies employed in these experiments are supported by literature which shows that motor units can fire at the upper bound of our experiments^18, 19^. The activity-dependent calcium signals were again acquired by ramping the frequency up and down to test for hysteresis effect. As the data shows, no significant hysteresis was apparent.

The linearity of calcium fluorescence with stimulus frequency that we observe is in accordance with the linear characterization derived in mathematical modeling by Helmchen *et al*.^20^ of action potential evoked calcium in pyramidal neuron dendrites. Taken with the assumption that the activity dependent calcium increase comes from influx of action potential-associated calcium ‘quanta’, and not calcium induced calcium intracellular release, as our data indicates (below), these observations support the claim that calcium has been monitored in the linear buffering range of the indicator, rather than in its saturation range. In addition, it’s likely that we’ve not outreached the lower concentration (linear) regime of dye indicator buffering, as the intracellular baseline calcium concentration (~100 nM) is well below the indicator Kd(~380 nM)^21, 22^, and fluorescence transients from that baseline have occupied a small portion of the available dynamic range of the indicator (<0.3 and 14 respectively). Another piece of evidence supporting that these experiments were performed in the linear indicator buffering range is the data of linear frequency modulation for non-steady-state stimuli. In addition to the linear frequency dependent data presented in Fig. 3, in which amplitudes were taken from sustained, steady-state signals, the frequency-amplitude relationship for shorter, non-steady-state stimuli of 0.5 s was also observed to be linear (data not shown). Frequency modulated data collected in different points of the saturation curve shown in Fig. 2b would be not be expected to both be linear, if the dye was in a non-linear (saturating) regime of calcium buffering.

**Figure 4:**
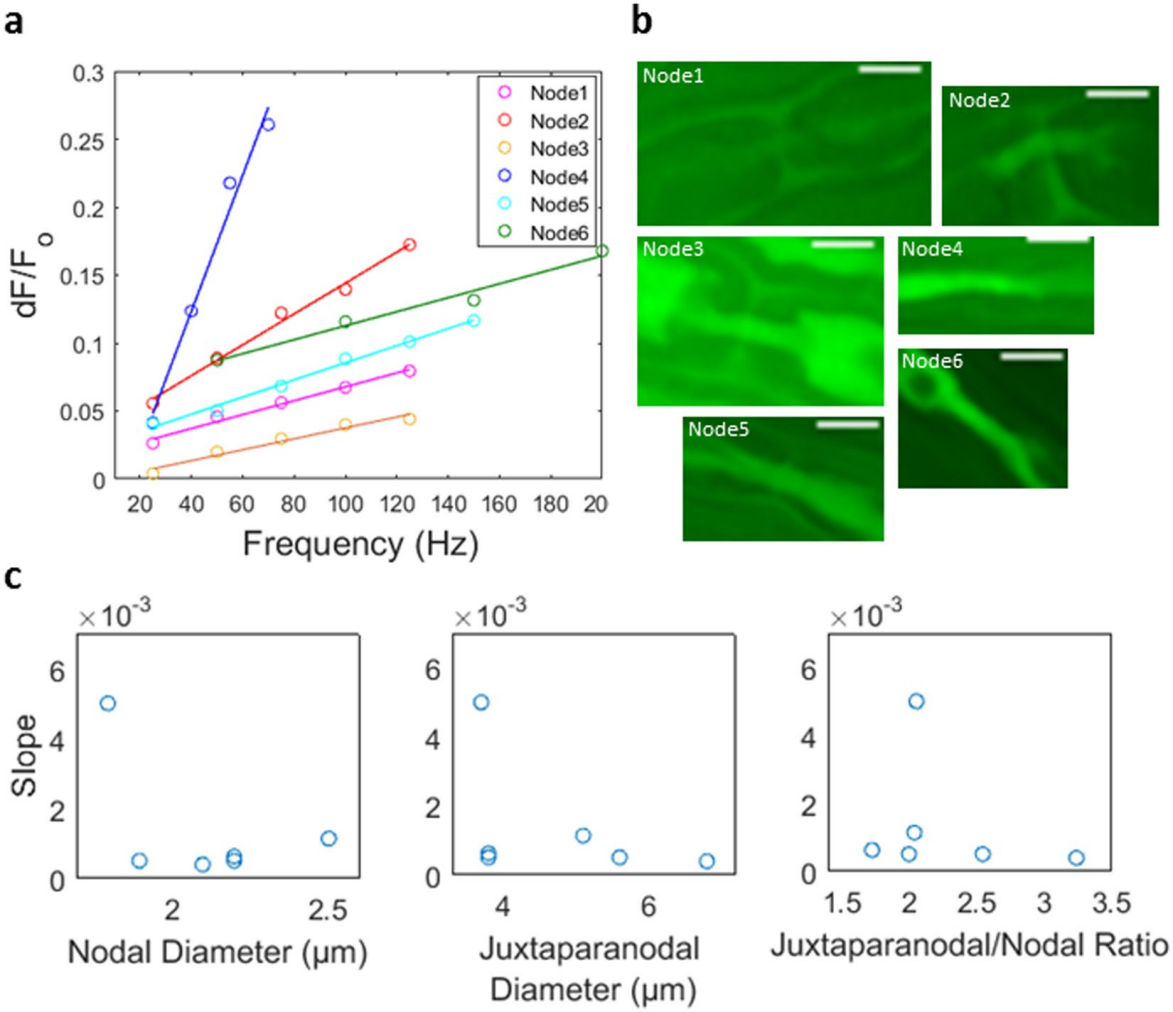
Comparison of frequency modulation slopes to nodal size (**a**) Frequency modulation slopes from nodes of six independent nerve sample. The nodal dF/F_o_ vs. frequency traces are the ramp up and down (two sweep) means with the exception of Node4 and Node6 which are single sweep traces. (**b**) Images of each axon node (scale bar 5 μm). (**c**) Slopes versus nodal diameter, juxtaparanodal diameter, and juxtaparanodal/nodal ratio do not reveal correlation between nodal size and slope.

The frequency modulation slope of a given node could not be correlated with its size, based on analysis from six independent samples of nodal diameter, juxtaparanodal diameter, and juxtaparanodal/nodal ratio (Fig. 4).

The modulation of calcium amplitude by stimulus frequency presented here is of important significance to the objective use of these signals as read-out in a neural interface, as it suggests that the neural ‘intensity’ can be inferred by the linearly proportional calcium accumulation in nodes of Ranvier.

### Signal Modulation by pulse-train duration

Another important property of the calcium signal is its modulation by pulse train *length*. Figure 3c shows calcium fluorescence responses to constant frequency action potential trains of graded duration, illustrating the fluorescence persisting over the duration of stimulus, with amplitude holding constant. Mean decay constants were not significantly different among any of the four pulse durations over three datasets from three independent samples (ANOVA, F = 0.82, df = 8, p = 0.516).

### Calcium transients are dependent on extracellular calcium

**Figure 5:**
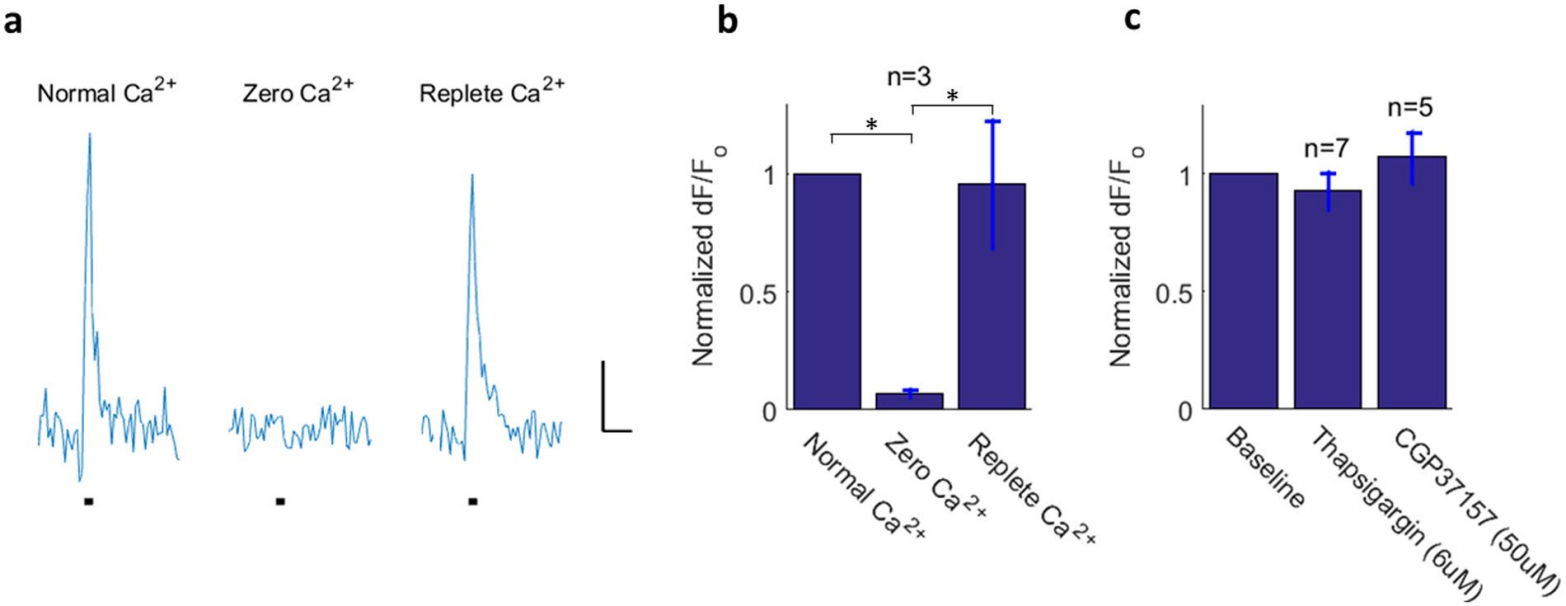
Extracellular calcium removal causes abolishment of axonal calcium-fluorescence transients. (**a**) Nodal calcium response to 20 action potential bursts (100 Hz) in normal calcium, zero-calcium, and replete calcium. Single sweep recordings are shown. *Scale bar: 1* *s & 3% fluorescence change*. (**b**) Results are summarized for three independent experiments indicating unequal means between normal calcium and zero calcium states (p = 0.014) and between zero calcium and replete calcium states (p = 0.017) (ANOVA with Tukey-Kramer test for multiple comparisons, F = 11.04, df = 6). (**c**) Application of the ER Ca^2+^-ATPase blocker thapsigargin (6 μM) and the mitochondrial Na/Ca exchanger blocker CGP37157 (50 μM) did not significantly reduce activity-dependent nodal calcium amplitude.

To investigate signal dependence on trans-membrane calcium influx, calcium was removed from the bath solution. Ca^2+^ was replaced with Mg^2+^ and 1 mM EGTA was added for the calcium-free buffer. The action potential-elicited calcium signals were largely abolished within 15 minutes in zero-calcium environment and returned to near baseline with calcium replenished (Fig. 5a,b). These data strongly suggest an influx component to the signal.

### Insignificant signal contribution by ER and mitochondria

To investigate the possibility that calcium influx or another activity-dependent signal could initiate calcium release from intracellular organelles, signal contribution from ER and mitochondria was tested. Thapsigargin^23^, a selective blocker of ER Ca^2+^ - ATPase was applied. We did not observe a statistically significant reduction in calcium signal amplitude in response to this blocker (paired-sample t-test, p = 0.423) (Fig. 5c). To test the mitochondrial contribution, the drug FCCP was first tested. This drug blocks the electron transport chain and thus effectively shuts down mitochondria. However, measurements of the compound action potential showed that FCCP abolished the action potential, so it could not be used to study the effect of mitochondrial inhibition on activity-dependent calcium. Instead, the mitochondrial sodium-calcium exchanger (mNCX) inhibitor CGP37157^24^ was employed. As with thapsigargin, application of this inhibitor did not result in significant reduction of activity-dependent calcium amplitude (paired-sample t-test, t = 0.874, df = 4, p = 0.431) (Fig. 5c).

### T-type Ca_V_ –channel inhibition blocks subset of nodal signals

**Figure 6:**
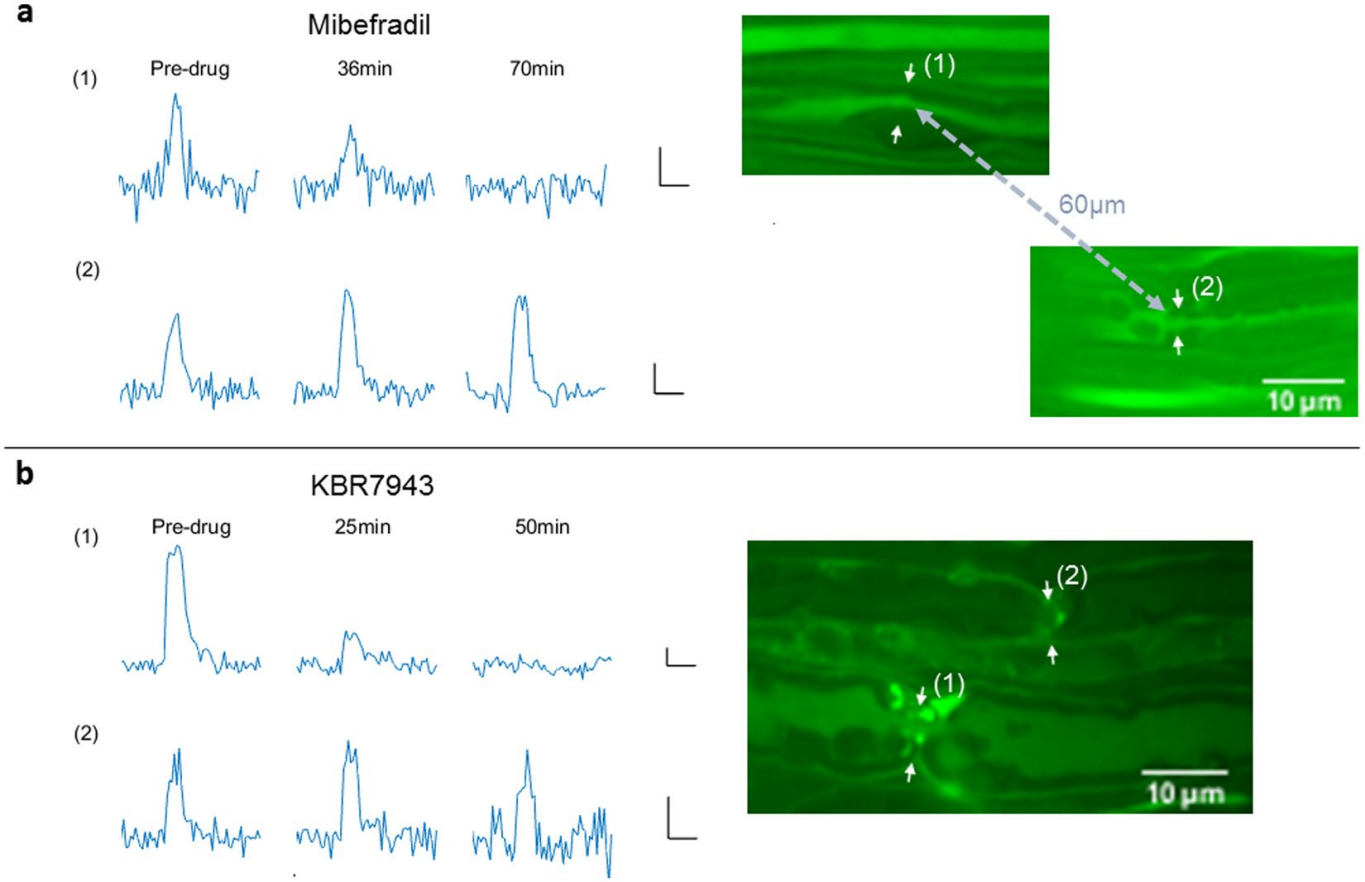
Nodes in close proximity show differential response to blockers. (**a**) Signals of two adjacent nodes in the same mibefradil-treated nerve sample show the blockage of signal in node (1) and the persistence of signal in node (2). (**b**) Two adjacent nodal signals in a KBR7943-treated nerve similarly shows a differential drug effect, with nodal signal (1) blocking and nodal signal (2) remaining unblocked. Single sweep recordings are shown. *Inset scale bars are 1* *s*, *and 3% dF/F*_*o*_.

**Figure 7:**
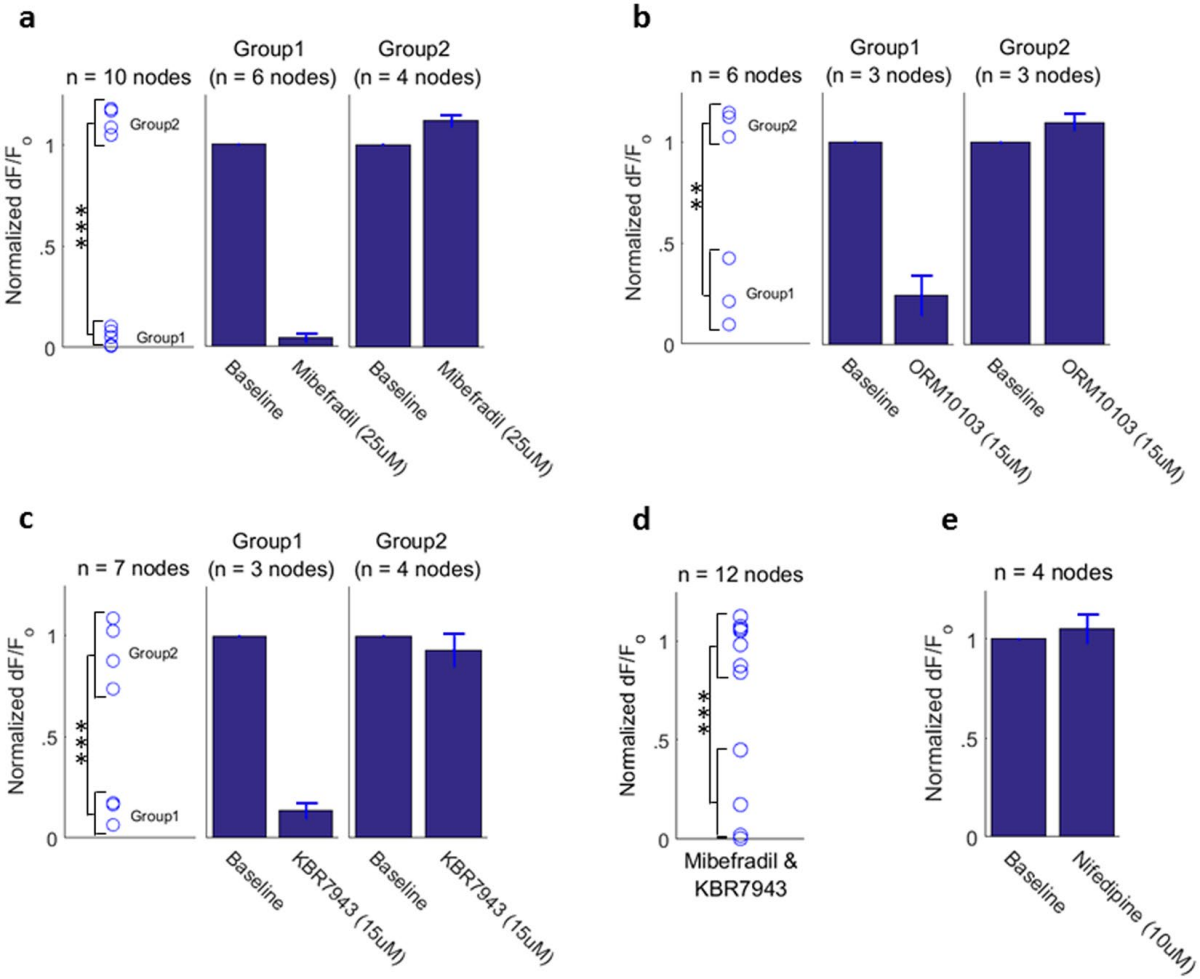
Ca_V_ channel and NCX blockers show differential inhibition of activity-dependent calcium response. (**a**) T-type Ca_V_ channel blocker mibefradil (25 μM) nearly abolishes the calcium signal in some nodes while other nodes are unblocked. (**b**) NCX inhibitors ORM10103 (15 μM) and (**c**) KBR7943 (15 μM) also show differential blockage: a set of nodal signals are significantly diminished while others show little or no blockage. (**d**) Co-application of mibefradil and KBR7943 shows similar results as seen for single drug application. (**e**) Activity-dependent calcium signals were not blocked or diminished by 10 μM nifedipine (L-type inhibitor). (Recordings from each data panel came from 2–4 mouse nerves).

Calcium-handling mechanisms capable of facilitating the apparent trans-membrane influx were studied. The T-type Ca_V_ –channel blocker mibefradil (IC_50_ = 2.7 μM)^25, 26^ was applied (25 μM) and caused the near abolishment of some nodal signals while others appeared unblocked. This observation was substantiated by instances where within the same region of a nerve sample some nodal calcium signals were blocked while other nodes in close proximity remained unblocked (Fig. 6a). A number of nodes were monitored under mibefradil application (n = 10), and the data appear to show two distinct groups of responses (Fig. 7a), one of which is unaffected by the drug, and the other of which is significantly diminished by T-type Ca_V_ inhibition. Among the ten nodes tested, six displayed blockage, and four did not, i.e. the data was split roughly into equal groups. A two-sample t-test indicated separate group means (t = −32.5, df = 8, p = 9e-10). The involvement of L-type voltage-gated calcium channels in nodal calcium influx was tested with nifedipine (10 μM), a well-known blocker of L-type calcium current^27^. Incubation in nifedipine, however, did not diminish the calcium signal amplitude (paired-sample t-test, t = 0.747, df = 3, p = 0.509) (Fig. 7e). While the T-type Ca_V_ blocker mibefradil also has mild selectivity for L-type Ca_V_, inhibition due to mibefradil was attributed to T-type channels since L-type block with nifedipine was without effect.

### Na-Ca Exchanger inhibition blocks subset of nodal signals

The plasma membrane sodium-calcium exchanger (NCX) was also investigated as a source of calcium influx. At resting membrane potential and ionic gradients, the NCX operates in the forward mode which imports Na^+^ into the cell down its concentration gradient while pumping Ca^2+^ out of the cell at a stoichiometry of three to one respectively. However, during membrane depolarization and intracellular Na^+^ elevation (i.e. during the action potential) the NCX can operate in reverse mode, pumping Na^+^ out and Ca^2+^ in. A primary example of reverse-mode NCX operation occurs as an integral part of the cardiac action potential^28^.

Two specific blockers of the NCX were employed to test its contribution. KBR7943^29^ and ORM10103^30^ are potent inhibitors of the reverse mode NCX (IC_50_ = 0.3 μM and 1 μM respectively). The results for each drug were similar; two groups of responses were present. Some nodes were either mildly affected or unaffected, while others displayed a significant degree of blockage (Fig. 7b,c). As with the mibefradil-exposed samples, nodal responses were statistically separated into two groupings, as indicated with a two-sample t-test (KBR7943: t = −8.22, df = 5, p = 4e-4, and ORM10103: t = −8.29, df = 4, p = 0.001). An example of the differential response to this inhibition is illustrated in Fig. 6b.

From these data we hypothesized that one set of axons exhibits a primarily T-type Ca_V_ – mediated calcium signal while another set has NCX – dominated calcium influx. In this scenario, co-application of both blockers should inhibit all, or at least more, nodes than with either drug alone. Mibefradil (T-type Ca_V_ inhibitor) and KBR7943 (NCX inhibitor) were applied in conjunction. However, the results of this experiment (Fig. 7d) did not support this hypothesis and require an alternate explanation. It was observed that no fewer nodal signals were diminished with drug co-application. While the grouping between ‘largely uninhibited’ and ‘significantly inhibited’ was less clear than single drug applications alone, the distribution exhibited bimodality, as tested with the Ashman D’s statistic (D = 5.22, where D > 2 indicates bimodality)^31^ and subsequent two-sample t-test (t = −9/05, df = 9, p = 8e-6). Collectively our data implicate the involvement of Ca_V,T_ and NCX in peripheral nerve activity-dependent calcium transients, but clear roles of these mechanisms and their allocation among axon type warrants future work.

## Discussion

Using an *in vitro* rodent nerve model with axon-loaded synthetic calcium indicator, we demonstrated and characterized action potential-elicited calcium signaling in the mammalian peripheral axon. This calcium signaling was localized to the node of Ranvier, a channel-dense and metabolically active region that facilitates saltatory conduction. Given the widespread signaling functions that calcium serves in the nervous system, and glial-axon signaling which is reported to occur at the node^32–36^, it is likely that this calcium signal has an important physiological role, and could potentially be involved in the development/maintenance of myelination.

Dynamic and mechanistic characteristics were investigated in this paper. The calcium signal was observed to arise at the node center, and propagate bi-directionally a short distance into the internode. Mean calcium wave propagation velocity and length constant of amplitude decay were calculated for a typical signal. Transients in calcium could be detected in response to a single action potential, while the calcium fluorescence amplitude in response to trains of action potentials rose sharply at low numbers of action potentials before approaching a steady state plateau with higher action potential trains. The steady state regime of a train-evoked signal, where calcium extrusion becomes balanced with unit influx, resulted in a relative ‘table top’ signal shape.

A pertinent characterization to neuroprosthetic application was that of frequency modulation. Since the force/velocity of a motor command is neurally dictated by the action potential frequency, it is a useful feature of a motor control signal to modulate its amplitude in proportion to this stimulus frequency. Thus, driving a prosthetic actuator in proportion to frequency-modulated calcium could enable intuitive prosthesis manipulation. As expected, the amplitude of calcium fluorescence summated with the number of action potentials in the stimulus (pulse train frequency). In agreement with studies of dendritic calcium accumulation^20, 37^, the amplitude-frequency relationship was linear. This finding fits with mathematical modeling that shows a linear dependence of mean amplitude on action potential frequency, given a consistent unit calcium quantity added per action potential. The frequency versus amplitude dependence of a node did not appear to be correlated with axon size; varying slopes may be attributed to differing unit calcium influx and/or variable dye/buffering conditions.

The observation of signal modulation by stimulus duration further establishes this type of signal command as a direct and intuitive neural read-out and control method. The signal persists for the duration of an action potential train and decays upon stimulus cessation; thus the temporal bounds of the activity is translated well by the calcium signal.

Pharmacological experiments were performed to investigate the mechanisms and pathways of the calcium elevation. This work suggests that the calcium signal arises from transmembrane influx rather than intracellular release from ER or mitochondria, as blockers of calcium handling mechanisms on these organelles did not significantly decrease the signal amplitude. Specifically, data presented here implicate the involvement of T-type Ca_V_ and NCX. These findings are consistent with the study in CNS Purkinje axons^14^ in which T-type Ca_V_ are found to be responsible for nodal calcium influx. Zhang & David^17^ also point to T-type Ca_V_ and NCX, and suggest that these two proteins are co-localized at the node with each contributing to the increase in calcium. Immunohistochemical reporting of T-type Ca_V_ channels in peripheral nerve is limited to nociceptors^38^, which are small-diameter fibers that are unmyelinated or thinly myelinated. Rose *et al*.^38^ showed Ca_V_3.2 immunoreactivity restricted to nociceptive axons. We have not recorded from unmyelinated axons, which are typically less than 1 μm in diameter and are much smaller than the axons we studied. Little is known about the distribution of these channels in larger peripheral axons from studies using immunolabeling, perhaps due to the poor characterization of commercial antibodies specific for its subtypes or for pan-specific antibodies. The distribution of the three NCX isoforms is also unknown in peripheral axons, again due to limited characterization of existing antibodies.

In the present experiments, the fact that some axons showed diminished signal in response to inhibitors while others did not could suggest a non-homogeneous distribution of these mechanisms. As such, it is worth pointing out experimental differences. The study by Zhang & David employed microelectrode dye injection, studying single large diameter (>5 μm) axons of the phrenic nerve. Furthermore, the employed concentration of the T-type Ca_V_ blocker mibefradil was likely below the IC_50_ for these channels^26, 39^, and thus, the data may underestimate contribution of T-type Ca_V._ The experiments of this paper do not distinguish between motor and sensory axons; thus, it is possible that these signals are restricted to motor or sensory axons. Zhang & David concluded that they were recording from some motor axons, but most of their recordings were from functionally unidentified axons. This uncertainty may underlie different classes of responses in the pharmacological studies presented here.

In the current study, nodal responses with inhibitors of both these pathways fell into two groupings: signals from some nodes were significantly diminished or blocked by drug application, while others were largely unblocked. Nodal and juxtaparanodal diameters were not statistically different between these groupings (data not shown). Co-application of T-type Ca_V_ and NCX blockers did not inhibit all signals as was hypothesized, but rather yielded a distribution of response amplitudes ranging from unblocked to blocked. This distribution of responses did appear to have grouping similar to the single drug data, and indeed was determined to be bi-modal, per the Ashman’s D statistic for bi-modality. Taken together, this data may suggest that the distribution of peripheral nerve axons studied do not have a single mode of calcium handling, nor is it likely that there are simply two functional classes of calcium signaling (i.e. T-type Ca_V_-dominated and NCX-dominated). Rather, the distribution of calcium handling mechanisms may be even more heterogeneous. A potential explanation for the observation that some nodal signals remained unaffected with drug co-application is that there is a third mode of calcium elevation. Future work will be required to achieve a more complete understanding of the heterogeneous axon distribution as it relates to calcium signaling mechanisms.

In summary, we have characterized an activity-dependent calcium fluorescence signal in axon nodes of Ranvier. By demonstrating that neural activity in a peripheral nerve can be transduced to an optical signal, we take a step forward in establishing feasibility of optogenetic read-out as a means to interface in the peripheral nervous system. We have demonstrated a calcium-dependent optical signal which can encode neural activity of individual axons over a wide range of action potentials. Indeed separate, unpublished work carried out by our laboratory has demonstrated real-time graded motor control of a prosthetic digit by this calcium signal in an *in vitro* preparation.

This work was primarily driven by the desire to interface with residual nerves of persons with amputations to provide axon specific prosthesis control. However, such an interface has the potential for much broader impact. The field of therapeutic neuromodulation further establishes the necessity for precise neural communication. The concept of treating, diagnosing and studying visceral diseases with the modulation of peripheral nerves such as the vagus nerve has received significant attention. As a recent review in this field of bioelectronic medicine points out^40^, the advancement of neural interfacing technology is essential to enable such strategies.

Future work will involve genetically expressed calcium sensors such as GCaMP, which have been used to study neural circuitry in *in vivo* animal models^41–44^, including their incorporation with deep tissue two-photon imaging^45^. The latest generation, GCaMP6^41, 42^, achieves reliable single action potential detection, with robust signal brightness. Red-shifted GCaMP analogues could potentially provide the advantages of better spectral separation for dual-wavelength systems and greater optical tissue penetration. Latest generation red protein calcium indicators such as the mRuby-based RCaMP, and the mApple-based R-GECO, are beginning to rival GCaMP in achievable signal amplitude, becoming a more viable option^46, 47^. The exploitation of this mechanism for useful neuroprosthetic control and therapeutic application will depend on optical tissue penetration constraints and the development of a cuff-mounted device that can optically scan the neural tissue appropriately. The recent and ongoing development of miniaturized optical fiber–coupled microscopes^48, 49^ may soon enable fast three-dimensional scanning *in vivo*, with the integration of electro-wetting lenses enabling rapid depth-dimension scanning^50^.

## Methods

### Nerve Preparation

The sciatic nerve and its tibial nerve branch are excised from adult wild type mice, and loaded from the tibial end with a synthetic calcium indicator (2 mM Calcium Green-1 Dextran, ex/em = 506/531 nm) and a calcium insensitive dye (1 mM Rhodamine-B Dextran, ex/em = 570/590 nm) dissolved in a buffer containing 130 mM KCl and 30 mM MOPS, pH 7.2 (Supplementary Figure 1). (Rhodamine-B is co-applied to provide bright axon labeling, as the baseline cytosolic Calcium Green-1 signal is relatively low). The tibial end is freshly cut in a zero-calcium buffer (Mouse Saline with Ca^2+^ replaced with Mg^2+^, and 1 mM EGTA added) to ensure open axon cylinders before being suctioned into a tight-fit electrode with the dye buffer to facilitate longitudinal axonal dye-loading via diffusion and/or axoplasmic transport. The suction electrode on the tibial nerve also serves to record electrical activity within the nerve. The sciatic end of the nerve is drawn into a suction electrode for electrical stimulation of compound action potentials (CAPs). The nerve sample is stored initially in a mouse saline solution (in mM: 126 NaCl, 5 KCl, 1.8 CaCl_2_, 1 MgCl_2_, 10 MOPS Buffer pH 7.2, 30 glucose) during experimental preparation and is perfused with a modified Tyrode’s solution (in mM: 126 NaCl, 3 KCl, 2 MgSO_4_, 20 NaHCO_3_, 1.2 NaH_2_PO_4_, 2 CaCl_2_, 30 glucose) bubbled with 95%0_2_/5%CO_2_, for the duration of the two hour dye loading period and subsequent imaging/electrophysiology.

The use of animals was approved by the Institutional Animal Care and Use Committee (IACUC) at the University of Colorado Health Sciences Center, with accreditation by the Association for Assessment and Accreditation of Laboratory Animal Care (AAALAC). All experiments were performed in accordance with IACUC regulations and approved protocol.

### Electrophysiology

CAPs are generated and recorded throughout the experiment (MultiClamp 700B Amplifier, Axon Instruments, PCLAMP10 Software) using 50 µs square pulses to confirm and monitor nerve viability. The stimulation voltage threshold for maximum CAP amplitude is determined, and a modestly supra-threshold voltage is subsequently used to elicit calcium signals. CAP amplitudes were monitored throughout the duration of the incubation period, to confirm stable nerve health.

### Electrode and Chamber Fabrication

Electrodes were custom fabricated; capillary glass was heat extruded and the annulus custom-sized to the nerve diameter with a diamond scribe and fire polishing. The electrical leads were made from Teflon-coated silver wire, the ends of which were stripped and ‘chlorided’ using bleach. The chamber was designed in SolidWorks software and 3d-printed.

### Optical imaging/recording

Dye labeled axons were imaged in a region of nerve near the tibial recording electrode. The nerve was gently pressed to the optical glass of the chamber with low-tension silk strings attached to a small weight for imaging on an inverted microscope. Placement of the small ‘harp-like’ device did not affect the CAP. Fluorescence imaging was performed on a spinning disk confocal microscope (Intelligent Imaging Innovations, Marianas). A 515 nm laser line was used to excite the Calcium Green-1 and a 561 nm line was used for the RhodamineB tracer. Pixels were generally binned (2 × 2) to improve the frame read-out time for fast imaging. To record calcium transients, time-lapse images were acquired at 12–20 Hz, during which the nerve was stimulated by an electrical stimulator triggered via TTL pulses from the microscope. Fluorescence was imaged onto an EMCCD camera (Photometrics Evolve) through a 525/50 nm emission filter for the Calcium Green-1 channel and 617/73 nm for the RhodamineB channel. Images were collected with a 63X, 1.4NA oil-immersion objective lens. Image data were extracted from SlideBook6.0 software and analyzed and processed using custom MatLab script. Calcium signal traces and amplitude measurements presented are from single sweep recordings unless explicitly stated to be otherwise.

### Pharmacology

For experiments involving drug application, the epineurium was cut open to facilitate drug diffusion from the bath solution into the nerve bundle. Activity-dependent calcium signals were elicited by short bursts of action potentials; 50 action potentials or less at 100 Hz. Signals were generated by the stimulus at intervals of 2–5 minutes, and this interval was held constant within a given experiment. Once a stable baseline signal was established at one or more nodes, the drug was applied by bath exchange with drug-containing mouse saline. If a baseline calcium signal amplitude could not be established without deterioration, the node was not monitored for pharmacological effect. The activity-dependent calcium amplitudes were monitored for 35–70 minutes after drug addition.

### Statistics

Differences between group means were tested with two-tailed t-tests. A paired t-test was employed for non-independent data and a two-sample unpaired t-test was used for independent group data. For mean comparisons across more than two groups an ANOVA was used, along with the Tukey-Kramer method for multiple comparisons. Dataset bimodality was inferred per the Ashman’s D statistic^31^. Recordings from each categorical dataset came from a range of 2–6 independent mouse nerves with the exception of Fig. 2 in which the data was collected from three nodes in a single nerve.

## Electronic supplementary material

Supplementary Information

Supplementary Video 1
