# Supplementary material for: Optical Read-out of Neural Activity in Mammalian Peripheral Axons: Calcium Signaling at Nodes of Ranvier
Source: Sci Rep. 2017 Jul 18;7:4744. doi: 10.1038/s41598-017-03541-y (PMC5516017; doi:10.1038/s41598-017-03541-y)
Supplement: Supplementary file 1 — Supplementary Information [file 41598_2017_3541_MOESM1_ESM.pdf]

Optical Read-out of Neural Activity in Mammalian Peripheral Axons: Calcium Signaling at Nodes of Ranvier

Arjun K. Fontaine[1,], Emily A. Gibson[1], John H. Caldwell[2,3], Richard F. Weir[1,3]*
Departments of [1]Bioengineering and [2]Cell and Developmental Biology, University of Colorado – Anschutz Medical Campus
[3]Co-senior authors

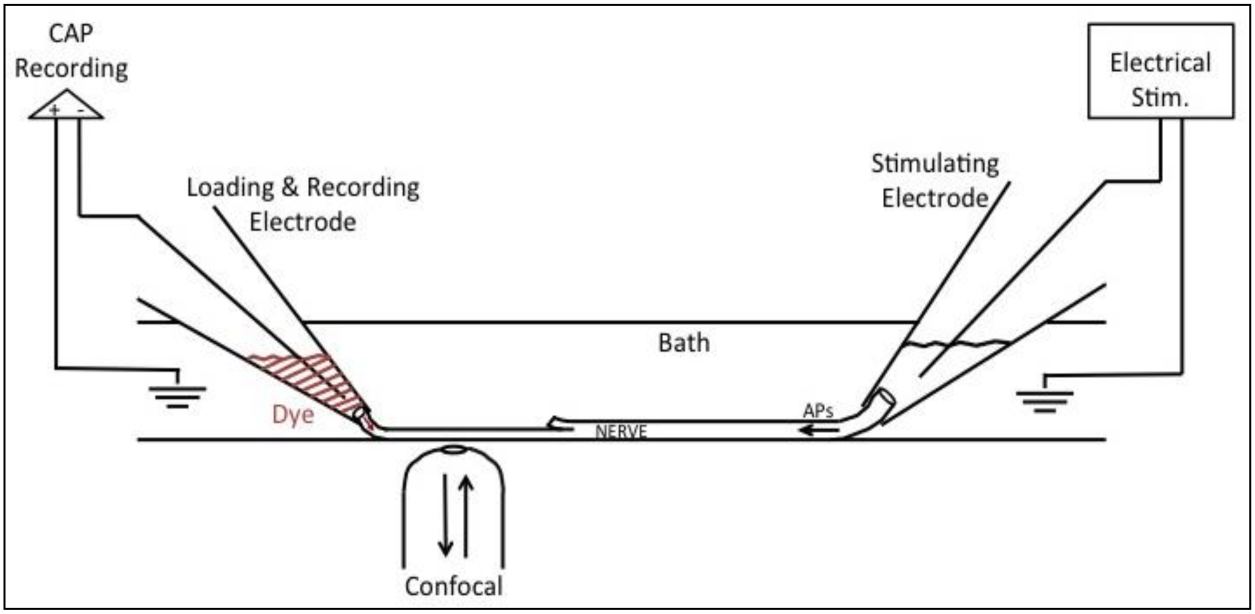

Supplementary Figure 1: Experimental configuration of nerve loading, electrophysiology, and confocal imaging, with custom fitted electrodes.

Supplementary Video 1 (attached file): Activity-evoked calcium response at an axon node of Ranvier to a burst of action potentials (50 @ 100Hz). ***Scale bar 4µm.***
